# A data navigation model to improve access to research data resources in clinical and translational science

**DOI:** 10.1017/cts.2026.10783

**Published:** 2026-07-01

**Authors:** Neena Thomas, Ayse Odabas, Soledad Fernandez, Lang Li, Julie Johnson

**Affiliations:** 1 Center for Biostatistics, The Ohio State University, Columbus, OH, USA; 2 Department of Biomedical Informatics; Center for Biostatistics, The Ohio State University, Columbus, OH, USA; 3 Department of Biomedical Informatics, The Ohio State University, Columbus, OH, USA; 4 Pharmacy, The Ohio State University, Columbus, OH, USA

**Keywords:** data navigation, clinical and translational research, secondary data, research data governance, decision support tool

## Abstract

Clinical and translational investigators increasingly rely on complex institutional and national data resources, yet barriers related to data discovery, governance, and access pathways remain common. To address fragmentation in data access, we piloted a Data Navigation Program within the Clinical and Translational Science Institute (CTSI) that established a trained Data Navigator as a centralized first point of contact for investigator data inquiries who provided individualized consultations, facilitated connections to data domain experts and honest broker services, and increased awareness of institutional data assets and regulatory requirements. To better characterize investigator needs, a CTSI-wide survey assessing data sources, governance, and training priorities was conducted in collaboration with the Clinical Translational Data Science (CTDS) Workgroup. Results demonstrated strong demand for structured guidance in data discovery and governance navigation. These findings informed refinement of the program, including development of the Research Data Source Match, a self-service decision-support tool implemented in REDCap that generates customized data access roadmaps based on investigator characteristics and data needs. During the pilot year, the Data Navigator conducted consultations addressing electronic health record (EHR), PCORnet resources, and government datasets. Integrating personalized navigation with scalable self-service tools may reduce barriers and support responsible data use in translational research.

## Background and rationale

The expanding availability of electronic health record (EHR) data, real-world data, and large-scale national research datasets has created new opportunities for clinical and translational research [1–4]. However, investigators frequently encounter challenges in identifying appropriate data sources, understanding institutional and regulatory requirements, and navigating complex data governance and approval processes [2,5–8]. These barriers can delay study initiation, limit feasibility assessments, and disproportionately affect early-stage investigators and researchers without formal informatics training [2,9,10].

The Clinical and Translational Science Institute (CTSI) at The Ohio State University, supported by an NIH Clinical and Translational Science Award (CTSA), provides essential infrastructure, resources, and services to facilitate clinical and translational research across The Ohio State University and Nationwide Children’s Hospital [11]. Although substantial expertise exists within specialized institutional units, such as informatics cores and data stewardship groups, this expertise is not broadly distributed across departments and research programs. As a result, investigators often struggle to determine where to begin or how to align scientific goals with available data assets and access pathways. Static documentation and ad hoc consultations are often insufficient to address these needs at scale.

## Data navigation program

### Initial implementation of the data navigator

To address these gaps, we piloted a Data Navigation Program within CTSI to centralize data-related support, reduce fragmentation, and align institutional data services with investigator needs. The program established a Data Navigator function and designated for this role a Research Scientist and data analyst within the Center for Biostatistics, Data Access Analysis and Coordination (DAAC) Division with expertise in clinical informatics and experience supporting electronic health record (EHR) derived PCORnet data requests [12,13], institutional EHR data workflows, and Epic-based research tools. The Data Navigator served as the first point of contact for investigators seeking guidance on research data resources, access pathways, and governance requirements. The primary goals of the program were to improve efficiency in early-stage project planning, increase awareness of institutional and national data assets, and promote responsible data use.

To support this role, the Data Navigator received additional training in institutional data resources, governance processes, PCORnet workflows, and Epic-based research tools, including Epic Clarity and Caboodle certification. Training also included participation in investigator consultations and ongoing mentorship from senior faculty and staff with expertise in clinical informatics, data resources, and governance.

As CTSI explored opportunities to improve investigator access to research data resources, this combination of technical expertise and investigator-facing experience provided the foundation for development of the Data Navigator role. The individual continued to serve as a data analyst within the Center for Biostatistics while also functioning as the CTSI Data Navigator, providing consultations, raising awareness of institutional and national research databases and tools, assisting investigators in navigating regulatory and institutional approval processes, connecting investigators to data domain experts, honest brokers, data analysts and biostatisticians and delivering educational presentations and outreach activities. Through this role, the program emphasized coordination and navigation rather than duplicating existing analytic or informatics expertise.

Although early consultations demonstrated demand for data navigation support, a more systematic assessment was needed to characterize investigator needs and priorities across experience levels and data science domains. Specifically, we sought to identify which data sources were most in demand, whether governance navigation or technical training represented the greater barrier, how investigator needs varied by role or discipline, or which services should be prioritized for scalability. To refine and strategically expand the program, broader institutional engagement was necessary.

### CTSI-wide data science needs assessment

To better characterize investigator priorities, a CTSI-wide survey was conducted under the guidance of the Clinical Translational Data Science (CTDS) Workgroup, a multidisciplinary CTSI team, during October and November of the pilot year. This REDCap survey was distributed electronically as a mass email to faculty, staff, and trainees via CTSI communication lists, reaching approximately 2,500 individuals of whom 230 individuals opened the survey invitation email. A total of 110 respondents completed the survey, corresponding to a 47.8% completion rate among email openers and an estimated overall response rate of 4.4% based on the email distribution list. Participation was voluntary and anonymous, and the Data Navigator contributed to survey design and dissemination.

Importantly, this survey was designed as a general data science needs assessment, not as a direct evaluation of the Data Navigation Program. Survey domains included interest in institutional and national data sources, governance and regulatory topics, data science training priorities, and preferred formats for support. The survey instrument is provided in the Supplementary Appendix to support replication.

Data science in this context encompasses clinical informatics, bioinformatics, biostatistics, epidemiology, artificial intelligence, implementation science, and bioethics. Clinical and translational research includes pre-clinical studies, human subjects, research (including clinical trials), epidemiology, and community-based research.

**Figure 1.**
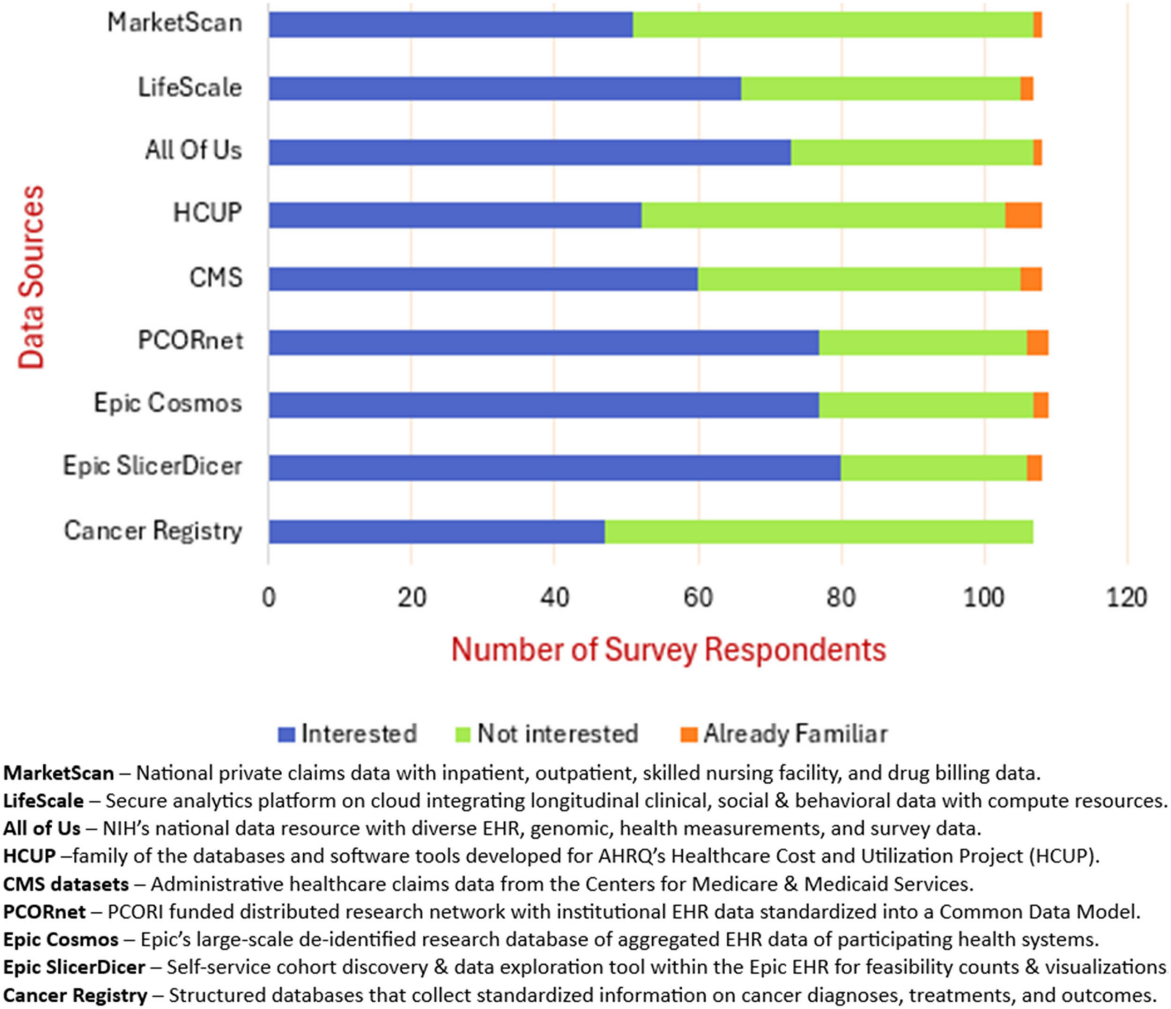
Investigator interest in training on available research data sources (CTSI survey, *n* = 110).

The CTSI survey respondents represented a broad range of academic ranks and research roles, including faculty, research staff, and trainees. The majority self-identified as having novice-level data science skills, underscoring the need for accessible, structured guidance. Figure 1 illustrates the results from this CTSI-wide survey.

Although the survey assessed broad data science interests, responses revealed particularly strong demand for guidance in identifying, evaluating and accessing available data sources, as well as navigating associated governance requirements. High levels of interest were reported for training on several commonly used research data resources, including Epic SlicerDicer (73%, 80/110 respondents), PCORnet (70%, 77/110) [12,13], Epic Cosmos (70%, 77/110), the All of Us Research Program (66%, 73/110), and datasets from the Centers for Medicare & Medicaid Services (55%, 60/110).

Regulatory and governance-related training needs were also prominent, including stewardship for EHR-based research (77 respondents) and governance for clinical trials and epidemiologic studies (73). Although survey respondents indicated substantial interest in a wide range of informatics and data science topics, including analytic pipelines, artificial intelligence applications, and programming skills, these areas were considered longer term workforce development priorities, rather than the primary focus of the pilot Data Navigation service.

In contrast, demand for structured navigation of data sources and governance processes represented an immediate and actionable need. Survey findings also aligned closely with consultation activity captured through a REDCap intake system. The most common requests included institutional EHR including Epic-based tools such as SlicerDicer, a self-service data visualization and reporting tool within the Epic EHR system, Centers for Medicare & Medicaid Services (CMS) datasets such as Standard Analytic Files (SAF) and other government datasets, and resources from PCORnet [12,13] mirroring the resources that generated highest survey interest.

These findings validated the importance of centralized data navigation, provided direction for program refinement and guided future program development, including self-service resources and consultation services to support early-stage planning and responsible data use.

## Refinement and expansion of the data navigation framework

Survey findings, combined with early consultation experience, led to expansion of the Data Navigation Program beyond its initial design. The refined framework integrates centralized intake and personalized consultation, governance-integrated decision support, scalable self-service tools, and integration with the CTSI website. These findings demonstrate convergence between expressed demand and real-world utilization of navigation services and correspond with the core functions of the Data Navigation Program and the Research Data Source Match tool, which integrates data access pathways with governance requirements.

### Research data source match tool

To extend the reach of personalized navigation services, we developed the Research Data Source Match, a branching decision-support tool implemented in REDCap [14]. This scalable, self-service decision-support tool helps investigators evaluate potential research data sources and associated governance pathways based on their scientific objectives, at their own pace. It is intended for structured, low-to-moderate complexity inquiries focused on identifying data sources and access requirements, whereas the Data Navigator provides individualized consultation for complex or multi-system requests requiring expert coordination.

Using conditional branching logic, the tool guides users through key decision points, including research intent, principal investigator (PI) status, and institutional affiliation (e.g., academic campus, affiliated medical center, or partner children’s hospital). Based on these inputs, the tool provides high-level guidance on governance considerations and next steps for access, for relevant data sources like institutional EHR data, local and PCORnet resources [12,13], claims data, cancer data, genomics data, and other specialized registries and private data sources.

**Figure 2.**
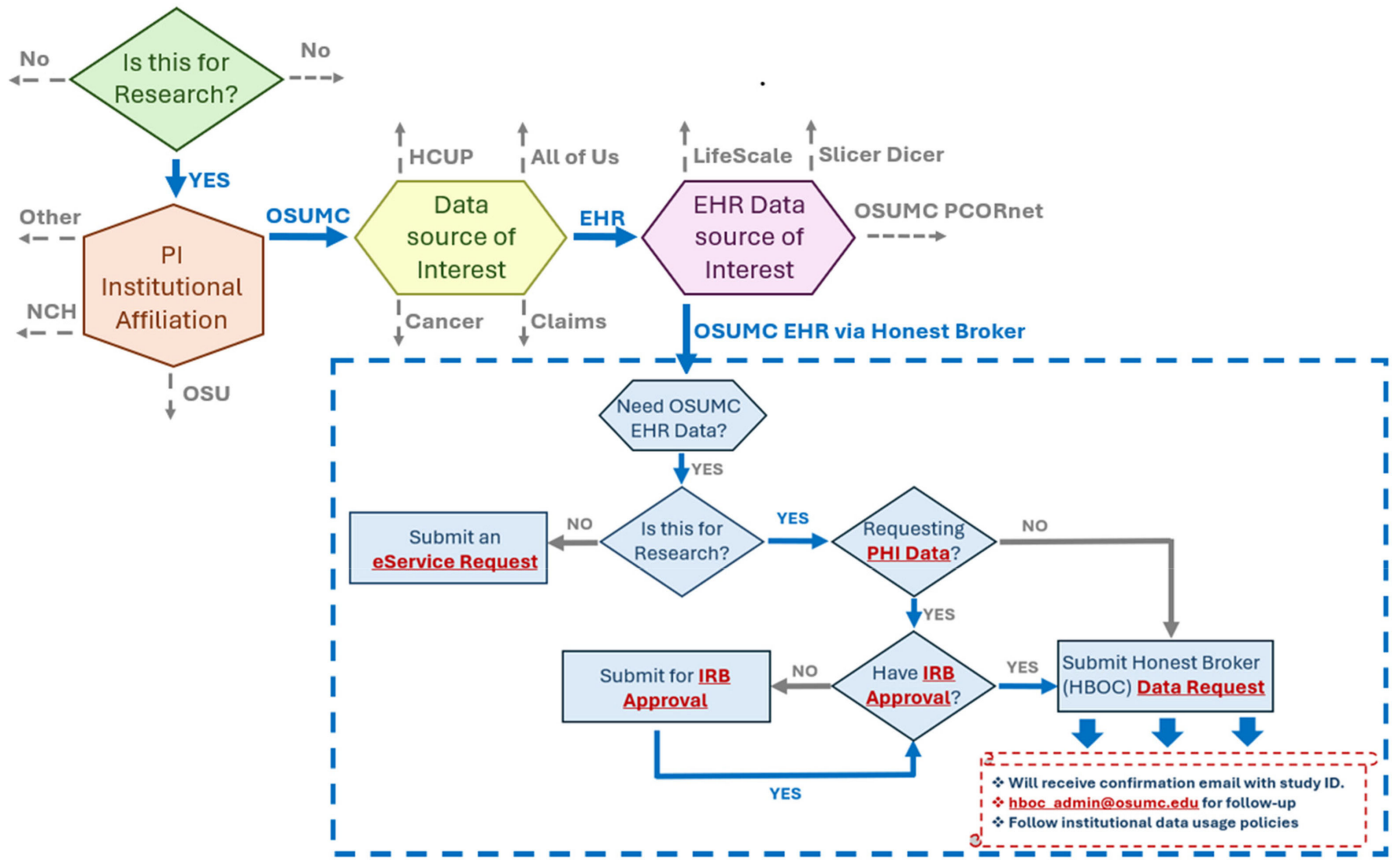
Example decision pathway in the research data source match tool.

Figure 2 illustrates an example pathway within the Research Data Source Match tool, highlighting branching logic based on investigator status, institutional affiliation, data source, and governance requirements. The solid blue arrows show accessing OSUMC EHR data through the honest broker process. Red text indicates embedded institutional request links, the blue dashed outline highlights the downloadable workflow view generated. Gray dashed pathways represent alternative data source (e.g., PCORnet) specific workflows not shown. Quality improvement activities follow separate institutional processes and are outside the scope of this research-focused tool.

The Research Data Source Match incorporates institutional data policies and regulatory principles related to data classification, security responsibilities, and reporting obligations. By presenting this information at the point of inquiry, the tool empowers investigators to independently explore feasible data options while reinforcing responsible data stewardship [3]. Embedding this tool within the CTSI website increased visibility and scalability while preserving expert engagement for high-complexity cases. Users can generate customized roadmaps tailored to their institutional affiliation and data needs and save these workflows for later reference. This approach extends the reach of the Data Navigator, enabling more investigators to access guidance efficiently without waiting for a one-on-one consultation, while also supporting early-stage planning and reproducible research workflows. Investigators with complex or ambiguous needs are directed to the CTSI Data Navigator for further consultation. Prior to implementation, tool pathways were reviewed by data stewards, honest brokers, and other institutional stakeholders to ensure alignment with current data access and governance requirements. Formal utilization metrics were not yet available at the time of manuscript preparation and will be evaluated in future program assessments.

**Figure 3.**
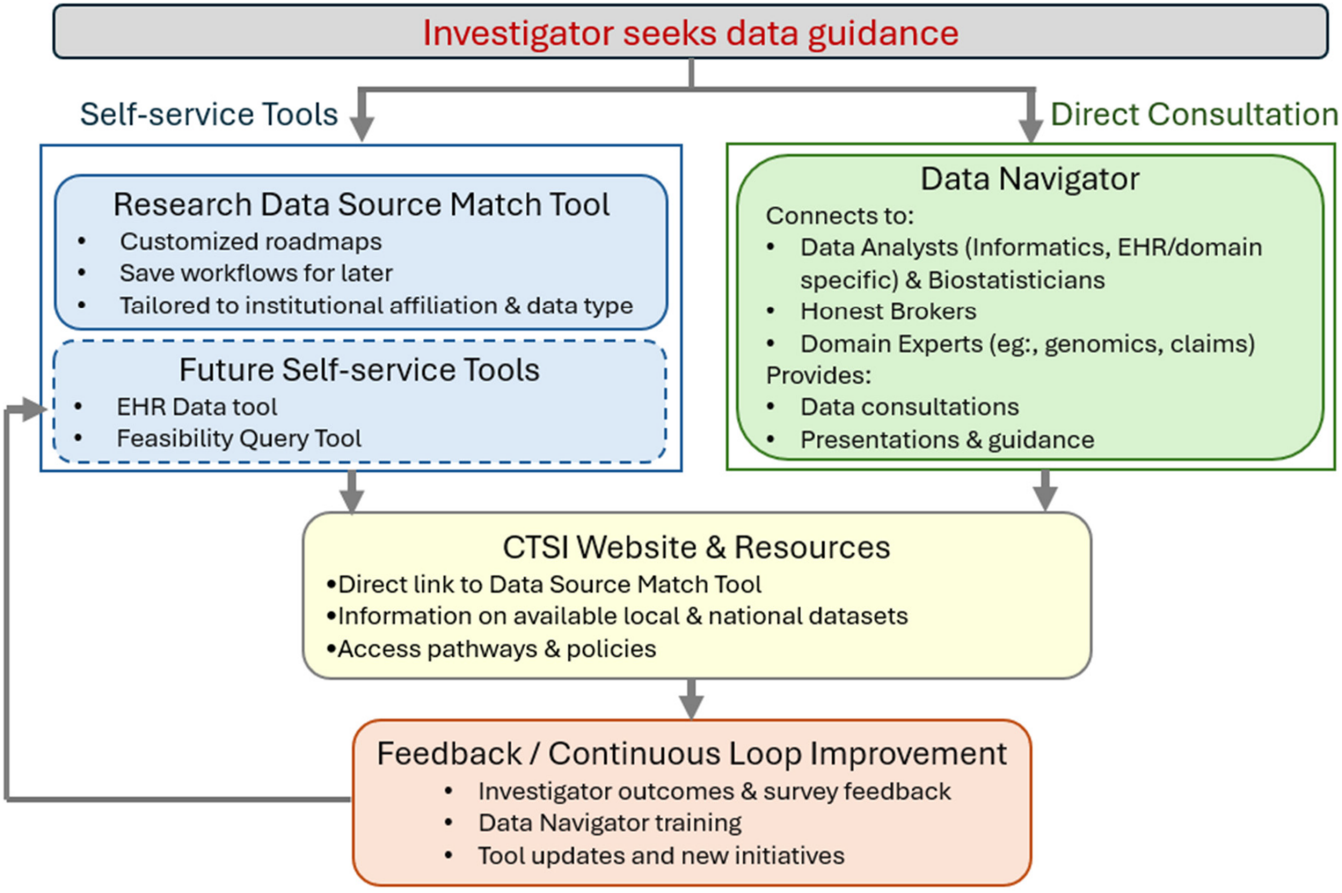
CTSI Data navigation program overview.

Figure 3 illustrates the overall structure of the Refined Data Navigation Program, including investigator pathways, available tools, consultation support, and the continuous feedback loop that informs program evolution.

To increase awareness and access, the institutional CTSI website was updated with descriptions of local and national datasets, access pathways, and direct links to the Research Data Source Match tool, enabling investigators to explore potential data sources independently. By embedding the tool within the CTSI website, we increase visibility, streamline access, and further extend the reach of the Data Navigation Program beyond individual consultations. Awareness of the Data Navigation Program was promoted through CTSI newsletters, website content, educational workshops, departmental presentations, and investigator consultations.

From program launch in September 2024 through February 2026 (18 months), the Data Navigator conducted over 90 consultations across multiple colleges at The Ohio State University and the partner institution, Nationwide Children’s Hospital. These consultations addressed a wide range of investigator needs related to research data discovery, feasibility assessment, and governance navigation. Frequently discussed topics included access to institutional EHR data, national research networks such as PCORnet [12,13], and government datasets from the Centers for Medicare & Medicaid Services. This consultation activity highlighted the breadth of investigator demand for structured guidance in navigating research data ecosystems and informed continued refinement of the Data Navigation Program and associated self-service tools.

## Discussion

This Special Communication describes the implementation and refinement of a Data Navigation model within a CTSA Hub, which was designed to reduce early-stage barriers to data-driven clinical and translational research. The survey was not conducted primarily to evaluate the Data Navigation Program; rather, it was used to assess broad data science needs and to help define and refine the full implementation of the Data Navigator efforts. However, navigation-related demand emerged as a dominant and actionable theme. These findings reinforced the strategic value of centralized navigation and informed expansion of the program’s functions [10,11].

The Research Data Source Match tool represents a novel extension of traditional consultation-based support. Rather than replacing personalized navigation, the tool functions as a front-end triage and education mechanism, allowing investigators to explore data options independently while reserving individualized consultations for more complex cases. The hybrid model, combining personalized consultation with a governance-aware self-service tool, promotes scalability while maintaining responsible stewardship and coordination with existing analytic cores [3,13].

Importantly, the Data Navigation model complements existing informatics and analytic cores by emphasizing coordination, awareness, and responsible data use rather than duplicating technical expertise [14]. Our findings build upon prior CTSA-supported research navigation and consultation models that provide investigators with centralized access to research expertise and services. Previous reports have described successful approaches for coordinating investigator support through consultation services and facilitating connections with methodological collaborators [16,17]. For example, Pomann et al. highlighted the importance of structured mechanisms for identifying analytic collaborators within academic medical centers [17]. The Data Navigation Program complements these efforts by focusing upstream on research data discovery, governance navigation, and data access pathways before analytic collaboration begins. The Data Navigation Program extends these concepts by focusing specifically on identification of research data resources, navigation of governance requirements, and integration of a self-service decision-support tool to support early-stage project planning. Within the CTSI Clinical Translational Data Science (CTDS) Workgroup ecosystem, the Center for Biostatistics serves as a centralized “one-stop shop” for study design, biostatistical consultation, EHR systems, and data analysis services, while partners in the Department of Biomedical Informatics contribute specialized expertise in data architecture, and informatics methods.

The Data Navigation Program extends this centralized model upstream by focusing on data source identification, feasibility considerations, and governance navigation before analytic execution begins. By connecting investigators with appropriate data resources and expertise early in project development, the program reduces fragmentation, clarifies pathways for investigators, and promotes more efficient use of institutional research infrastructure.

By positioning navigation upstream of analytic execution, the program complements biostatistics and informatics services rather than duplicating them, creating a coordinated continuum from data discovery to analysis.

### Key features enabling program success

Several design features contributed to the early success and scalability of the Data Navigation Program. These elements reflect both operational experience during the pilot period and broader principles for supporting investigators navigating complex research data ecosystems. Key design implementation components, enabling factors, common challenges, and mitigation strategies identified during development of the Data Navigation program are summarized in Table 1[1,3,12,14].

**Table 1.** Key design features of the CTSI data navigation program Table 1 long description.

Design feature	Description	Implications for implementation
Centralized intake with distributed expertise	The Data Navigator serves as a single-entry point for investigator inquiries while leveraging existing institutional resources for specialized analytic and informatics support	Simplifies investigator access to resources without requiring consolidation of all services within one unit
Cross-domain expertise in the Navigator role	The Data Navigator possesses experience in clinical data systems, research workflows, and institutional governance processes	Enables efficient translation of research questions into feasible data access strategies
Governance-integrated guidance	Data navigation incorporates regulatory requirements, data stewardship policies, and institutional governance processes into early-stage consultation	Promotes responsible data use and helps investigators anticipate approval requirements and timelines
Scalable self-service decision support	The Research Data Source Match tool provides structured guidance through a branching decision-support interface implemented in REDCap	Extends the reach of navigation services while reserving individualized consultations for complex cases
Institutional collaboration and alignment	Effective operation requires coordination among biostatistics, informatics, regulatory offices, data stewards, and CTSI leadership	Supports consistent guidance across organizational boundaries and strengthens program sustainability

### Implementation challenges,barriers and mitigation strategies

Implementation challenges were accompanied by corresponding mitigation strategies that informed iterative refinement of the Data Navigation Program. Coordinating across multiple data governance bodies and institutional units required relationship-building and clarity regarding roles and responsibilities. Differences in approval workflows, timelines, and data classifications across data sources and partner institutions introduced complexity that had to be translated into clear guidance for investigators.

Investigator expectations also presented challenges. Many investigators approached the Data Navigator with assumptions regarding rapid EHR data access or broad availability of certain datasets. A core function of the program therefore involved education regarding regulatory constraints, data quality limitations, and realistic timelines. Balancing responsiveness with responsible stewardship required consistent communication.

Scope management was another important consideration. Because the Data Navigator role is positioned at the interface of data discovery and analysis, consultations occasionally expand beyond navigation into detailed methodological planning or analytic execution. Clear delineation between navigation services and downstream analytic support provided by Center for Biostatistics and Department of Biomedical Informatics was necessary to maintain role clarity and sustainability.

Sustainability and maintenance represent ongoing considerations. The REDCap-based decision-support tool requires periodic updates to reflect evolving data policies, governance requirements, and available datasets. Scaling the program beyond a single Navigator will require attention to staffing models, training pipelines, and integration with existing CTSI service structures. The training approach described in this program, combining foundational instruction, certification in relevant data systems, mentorship, and participation in investigator consultations, provides a framework for onboarding future Data Navigators. Cross-training and documentation of workflows may further reduce the impact of personnel turnover and support continuity of services. This implementation also has several limitations. The program was implemented at a single CTSA hub, and while there may be institution-specific data resources and data access policies, the broad concepts of this program could be readily implemented at other CTSA Hubs/institutions. Survey data were self-reported and there is likely bias in the survey results based on those who chose to respond. Downstream outcomes such as grant submissions, funding success, or research productivity were not assessed during the pilot period. Future evaluations will examine longitudinal outcomes and scalability across institutional contexts.

Collectively, these experiences informed a practical implementation roadmap for other institutions, emphasizing centralized intake, clear role delineation, governance-aware navigation tools, stakeholder engagement, cross-training, and ongoing maintenance of institutional knowledge resources

### Implications and future directions

The Data Navigation Program offers a practical and replicable framework for CTSA hubs seeking to reduce fragmentation in research data access and support investigators navigating increasingly complex research data ecosystems. Our experience suggests that effective data navigation does not require consolidation of all analytic services within a single unit. Rather, it depends on structured coordination, clear role delineation, staffing by an individual with sufficient data access, use and governance knowledge, and alignment across existing institutional resources.

Institutions aiming to adopt a similar approach should tailor navigation models to local data ecosystems, and available expertise. Foundational elements include (1) a Data Navigator with cross-domain expertise in data science, clinical informatics, and governance; (2) strong partnerships among biostatistics, informatics, and data stewardship units; and (3) leadership support for centralized coordination. Rather than requiring consolidation of analytic services, the model emphasizes strategic alignment and communication across existing units. The specific scope of navigation services may vary across institutions. In some settings, data resource navigation and connections to quantitative collaborators may be integrated within a single program, whereas in others these functions may remain separate. The core principles described in Table 1 are broadly applicable across institutions, although implementation should be adapted to local organizational structure, available expertise, and complexity of data resources. Importantly, the Data Navigator role does not require a biostatistician; rather, it requires sufficient knowledge of institutional data resources, governance processes, and available support services to effectively connect investigators with appropriate expertise.

By integrating personalized navigation with scalable self-service tools and embedding governance considerations at the earliest stages of project development, the program supports efficient, responsible data use while reducing barriers for investigators, particularly those without formal informatics training. As CTSA hubs continue to expand their data ecosystems, navigation functions that integrate governance literacy, feasibility assessment, and analytic coordination may represent a critical layer of translational research infrastructure.

To further scale the Data Navigation Program and respond to needs identified through consultation activity and the CTSI-wide survey, several complementary initiatives are underway. These include development of an additional REDCap-based self-service decision support tool focused specifically on EHR data use for research, including data access pathways and regulatory requirements; a workshop series designed to build practical data skills and increase awareness of institutional resources; and a self-service cohort feasibility query tool to support early-stage study planning.

Although the CTSI-wide survey was not designed to evaluate the Data Navigation Program, its findings informed program refinement and expansion. The program directly addresses identified needs related to research data discovery, governance navigation, and connections to quantitative and informatics expertise through consultation services and investigator referrals. Early indicators of program utilization include more than 90 consultations conducted across multiple colleges and institutional partners during the first 18 months of implementation. Future evaluation will assess consultation volume, utilization of self-service tools, referral patterns, investigator satisfaction, and downstream outcomes related to research project development and data access.

Collectively, these efforts aim to enhance investigator autonomy during preliminary project development while preserving access to personalized consultation for projects requiring advanced coordination or methodological support. Future evaluation will examine longitudinal outcomes, including downstream grant activity, time-to-data-access metrics, and scalability across institutional contexts. A structured post-consultation feedback survey has been deployed to capture investigator-reported outcomes including whether consultations helped move requests forward, perceived clarity and usefulness of guidance, and downstream research progress. Response rates are currently limited, and data are not yet analyzed; therefore, outcome evaluation remains ongoing.

Importantly, the tools developed through this initiative were intentionally designed as low-burden, high-impact infrastructure that could be implemented rapidly within existing institutional ecosystems. While advances in artificial intelligence (AI), including conversational agents and automated knowledge systems, hold substantial promise for transforming research data navigation, such solutions are not yet sufficiently mature, institutionally integrated, or governance-aware to fully address the urgent and context-specific needs faced by investigators today. The demand for structured guidance in data discovery and governance navigation is immediate, and delaying implementation pending development of comprehensive AI-based systems would perpetuate existing access barriers [15]. Our current model therefore prioritizes pragmatic, scalable solutions that can operate within present institutional constraints. At the same time, the structured logic, governance integration, and workflow mapping embedded within the Research Data Source Match tool provide a conceptual and technical foundation that could be adapted to future AI-enabled navigation platforms as these technologies become operationally feasible and policy-aligned. In this sense, the present model serves both as an actionable solution for current needs and as an architectural precursor to more advanced, AI-supported data navigation systems.

## Supporting information

10.1017/cts.2026.10783.sm001 Thomas et al. supplementary material Thomas et al. supplementary material

## Supplementary material

The supplementary material for this article can be found at https://doi.org/10.1017/cts.2026.10783.

## Table 1. Long description

A table with four columns and five rows, including a header row. The columns are labeled Design feature, Description, and Implications for implementation. The rows detail various design features and their respective descriptions and implications. Row 1: Design feature, Centralized intake with distributed expertise; Description, The Data Navigator serves as a single-entry point for investigator inquiries while leveraging existing institutional resources for specialized analytic and informatics support; Implications for implementation, Simplifies investigator access to resources without requiring consolidation of all services within one unit. Row 2: Design feature, Cross-domain expertise in the Navigator role; Description, The Data Navigator possesses experience in clinical data systems, research workflows, and institutional governance processes; Implications for implementation, Enables efficient translation of research questions into feasible data access strategies. Row 3: Design feature, Governance-integrated guidance; Description, Data navigation incorporates regulatory requirements, data stewardship policies, and institutional governance processes into early-stage consultation; Implications for implementation, Promotes responsible data use and helps investigators anticipate approval requirements and timelines. Row 4: Design feature, Scalable self-service decision support; Description, The Research Data Source Match tool provides structured guidance through a branching decision-support interface implemented in REDCap; Implications for implementation, Extends the reach of navigation services while reserving individualized consultations for complex cases. Row 5: Design feature, Institutional collaboration and alignment; Description, Effective operation requires coordination among biostatistics, informatics, regulatory offices, data stewards, and CTSI leadership; Implications for implementation, Supports consistent guidance across organizational boundaries and strengthens program sustainability.

Navigate back to Table 1..
