# Supplementary material for: A data navigation model to improve access to research data resources in clinical and translational science
Source: J Clin Transl Sci. 2026 Jul 1;10(1):e117. doi: 10.1017/cts.2026.10783 (PMC13373600; doi:10.1017/cts.2026.10783)
Supplement: Thomas et al. supplementary material [file S2059866126107833sup001.pdf]

CTSI - CTDS Training Survey

As part of the Clinical and Translational Science Institute (CTSI) vision in Clinical and Translational Data Science (CTDS) to educate and support the translational research community and to empower research, the CTDS Workgroup would like to gather information and comments on Data Science and Data Source training opportunities of interest.

Please complete the survey below.

Thank you!

---

1. Please identify your data science training background and skills. (select one) *

- ◯ Novice
- ◯ Clinical and translational investigator with basic data science skills
- ◯ Clinical and translational investigator with advanced data science skills
- ◯ Data scientist

Novice - You have beginner level data science skills.

Clinical and translational investigator with basic data science skill - You can perform simple statistics analysis and data manipulation using Excel or R or other programs. While you are highly skilled on clinical and translational research and possess rich domain knowledge, you usually seek collaboration with data scientists for sophisticated informatics, statistics, and computer science analysis.

Clinical and translational investigator with advanced data science skill - You or someone in your lab are highly skilled in programming and conduct study design and data analysis by yourself. You are highly skilled on clinical and translational research and possess rich domain knowledge.

Data scientist - You and your lab conduct data science methodology research, or implement sophisticated informatics, statistics, and computer science methods for solving clinical and translational research questions. You are not a domain expert in clinical and translational research, but usually seek collaboration with clinical and translational investigators.

---

2. What is your current career stage? (select one) *

- ◯ Undergraduate student
- ◯ Graduate student
- ◯ Post-doc
- ◯ Research staff with a bachelor's or master's degree
- ◯ Research staff with a PhD degree
- ◯ Assistant Professor
- ◯ Associate Professor
- ◯ Professor
- ◯ Other

---

3. What data science training format fits you the best? (select all that apply) *

- ☐ In person hands-on workshop with introduction and real examples
- ☐ Introductory level seminar
- ☐ Semester-long individual course
- ☐ Graduate certificate

---

4. What data science training delivery approach fits you the best? (select one) *

- ◯ In person
- ◯ Online
- ◯ Hybrid

For Questions 5 to 10, please select your answers based on your level of interest.
How much are you interested in this particular data science training? (select one) *

5. How interested are you in learning more about the following informatics topics:

	Very interested	Moderately interested	Not interested because I know very well	Not interested because it is not related to my work	Not interested
Common healthcare data coding systems: such as ICD, CPT, SNOMED, LONIC, RxNorm, etc.					
Database models: hierarchical, network, star, flat, and relational models.					
Structured query language (SQL)					
Introduction to clinical databases, such as electronic medical record data and claims data.					
Clinical data integration and common data models.					
Data visualization design and development.					
Findable, Accessible, Interoperable, and Reusable (FAIR) principle in data management and data analysis.					

6. How interested are you in learning about the following data sources/tools:

	Very interested	Moderately interested	Not interested because I know very well	Not interested because it is not related to my work	Not interested
MarketScan - Inpatient, outpatient, skilled nursing facility, and drug billing data for millions of privately insured individuals across the United States.					

LifeScale - An institution-scale, honest-broker mediated, coded-limited instantiation of the OSU/NCH Epic Caboodle clinical data warehouse, loaded as parquet files and enriched with a diverse array of NMDH data, where the data is connected to a resourced Azure Databricks cluster governed by a joint agreement with NCH and OSU under a single, reliant-IRB protocol.	○	○	○	○	○
All of Us - Health data from a diverse group of participants from across the United States, where participants contribute to the program in many ways, such as by responding to surveys, sharing electronic health records, and providing bio samples.	○	○	○	○	○
HCUP (Healthcare Cost and Utilization Project) - Includes NIS, Nationwide Inpatient Sample, an all-payer inpatient database providing estimates of hospital inpatient stays, and NRD, National Readmission Database.	○	○	○	○	○
CMS (Centers for Medicare and Medicaid Service Standard Analytic Files) - Inpatient, outpatient, skilled nursing facilities, and hospice data for millions of publicly insured individuals across the United States.	○	○	○	○	○
PCORnet (The National Patient-Centered Clinical Research Network) - Coded limited common data model of electronic health records data from over 75 academic medical centers and health systems (grouped into eight Clinical Research Networks (CRNs)) spread all over the United States.	○	○	○	○	○

Epic COSMOS - Limited EHR data provided by participating organizations to Epic that is continuously updated including electronic health records from hundreds of millions of patients.	○	○	○	○	○
Epic SlicerDicer - A self-service tool that allows data exploration, search criteria refinement and ability to drill down into line level details, available on EHR directly or also as part of COSMOS environment.	○	○	○	○	○
James Cancer Registry - Institutional tumor registry which includes North American Association of Certified Cancer Registrars (NAACCR; compatible with Version 18) data elements and can be mapped to the EHR.	○	○	○	○	○

7. How interested are you in learning about the following bioinformatics data analysis pipelines:

	Very interested	Moderately interested	Not interested because I know very well	Not interested because it is not related to my work	Not interested
RNA-seq	○	○	○	○	○
ChIP-seq	○	○	○	○	○
Methylation	○	○	○	○	○
Genetic variant analysis	○	○	○	○	○
TCR-seq	○	○	○	○	○
Single cell RNA-seq	○	○	○	○	○
Spatial transcriptomics	○	○	○	○	○
Pathway analysis	○	○	○	○	○
Omics experimental design	○	○	○	○	○

8. How interested are you in learning about the following AI data analysis methods and applications:

	Very interested	Moderately interested	Not interested because I know very well	Not interested because it is not related to my work	Not interested

Natural language processing (NLP)	○	○	○	○	○
Drug development and pharmacology knowledgebase	○	○	○	○	○
AI for drug development	○	○	○	○	○
Generative AI such as large language models	○	○	○	○	○
EHR predictive analytical models	○	○	○	○	○
Image data analytics	○	○	○	○	○

9. How interested are you in training in the following data analysis and programming areas:

	Very interested	Moderately interested	Not interested because I know very well	Not interested because it is not related to my work	Not interested
Basic principles in machine learning and artificial intelligence in Python	○	○	○	○	○
Applied statistics in R	○	○	○	○	○
Clinical trial designs	○	○	○	○	○

10. How interested are you in the following data science related regulatory training:

	Very interested	Moderately interested	Not interested because I know very well	Not interested because it is not related to my work	Not interested
Data governance and stewardship for projects using electronic medical records	○	○	○	○	○
Data governance and stewardship for clinical trials and epidemiology studies	○	○	○	○	○
Bioethics in data science	○	○	○	○	○

11. Is there any other data source or data science training that you are interested in?

____
